# Comparison of Real-Time Water Proton Spectroscopy and Echo-Planar Imaging Sensitivity to the BOLD Effect at 3 T and at 7 T

**DOI:** 10.1371/journal.pone.0091620

**Published:** 2014-03-10

**Authors:** Yury Koush, Mark A. Elliott, Frank Scharnowski, Klaus Mathiak

**Affiliations:** 1 Department of Radiology and Medical Informatics, University of Geneva, Geneva, Switzerland; 2 Institute of Bioengineering, Ecole Polytechnique Fédérale de Lausanne (EPFL), Lausanne, Switzerland; 3 Department of Psychiatry, Psychotherapy and Psychosomatics, RWTH Aachen University, Aachen, Germany; 4 Center for Magnetic Resonance and Optical Imaging (CMROI), Department of Radiology, University of Pennsylvania, Philadelphia, Pennsylvania, United States of America; 5 JARA Translational Brain Medicine, Jülich - Aachen, Germany; INSERM U894, Centre de Psychiatrie et Neurosciences, Hopital Sainte-Anne and Université Paris 5, France

## Abstract

Gradient-echo echo-planar imaging (GE EPI) is the most commonly used approach to assess localized blood oxygen level dependent (BOLD) signal changes in real-time. Alternatively, real-time spin-echo single-voxel spectroscopy (SE SVS) has recently been introduced for spatially specific BOLD neurofeedback at 3 T and at 7 T. However, currently it is not known how neurofeedback based on real-time SE SVS compares to real-time GE EPI-based. We therefore compared both methods at high (3 T) and at ultra-high (7 T) magnetic field strengths. We evaluated standard quality measures of both methods for signals originating from the motor cortex, the visual cortex, and for a neurofeedback condition. At 3 T, the data quality of the real-time SE SVS and GE EPI R2* estimates were comparable. At 7 T, the data quality of the real-time GE EPI acquisitions was superior compared to those of the real-time SE SVS. Despite the somehow lower data quality of real-time SE SVS compared to GE EPI at 7 T, SE SVS acquisitions might still be an interesting alternative. Real-time SE SVS allows for a direct and subject-specific T2* estimation and thus for a physiologically more plausible neurofeedback signal.

## Introduction

Gradient-echo echo-planar imaging (GE EPI) is the predominant approach to assess localized blood oxygen level dependent (BOLD) signal changes [1]. Recent technological advances in the field of functional magnetic resonance imaging (fMRI) have made it possible to adapt GE EPI for use in real-time. Real-time fMRI can be used to provide a spatially localized neurofeedback signal based on the activation level of a region of the interest (ROI) [2]–[4], real-time brain-state classification [5]–[8], and connectivity-based neurofeedback [9]. Several studies have used real-time fMRI neurofeedback to train voluntary control over functionally specific brain areas, such as the motor and somatosensory cortices [10]–[13], the visual cortex [14], [15], the cingulate cortex [8], [16]–[19], the insula [20]–[22], the right inferior frontal gyrus [23], the amygdala [24]–[26], and the auditory cortex [27], [28]. Some studies even suggested potential therapeutic effects of real-time fMRI neurofeedback training in chronic pain disorders [17], Parkinson’s disease [29], tinnitus [28], schizophrenia [21], [30], and depression [31].

Depending on the magnetic field strength, the acquisition technique, and the echo time, the intra- and the extravascular components contribute differently to the functional BOLD signal changes. Generally, the use of ultra-high magnetic field strengths increases the signal-to-noise ratio (SNR) of GE EPIs, as well as the sensitivity and specificity of the T2* BOLD contrast [32], [33]. Higher magnetic field strengths also increase the SNR of the spin-echo (SE) EPI acquisitions which leads to improved localization of neural activity by targeting specifically microvasculature contribution to the BOLD signal [34], [35]. This is typically achieved by SE EPI acquisitions and T2 contrast at high spatial resolution, i.e. by suppressing extra- and intravascular components from large vessels that normally contribute to the BOLD signal at GE [34], [36]–[38]. Another difference between SE and GE techniques is that the maximum amplitude of the SE BOLD contrast is reached more quickly than that of the GE contrast. However, SE acquisitions usually have a lower SNR and a lower contrast-to-noise ratio (CNR) [35], [39], [40]. A disadvantage of ultra-high fields is that they are more prone to local field inhomogeneity, and thus require additional shimming adjustments and post-processing [41].

Functional SE single-voxel spectroscopy (SE SVS) has previously been employed for neurofeedback applications by using a point-resolved spectroscopy (PRESS) acquisition protocol to acquire the large water peak in the spectrum [42]–[44]. Compared to conventional metabolite quantification methods where the water peak is typically suppressed [45]–[49], SE SVS acquisitions allow for a direct estimation of T2* from the unsuppressed water spectrum [44], [50], [51]. More specifically, the T2* neurofeedback signal was estimated directly using free induction decay optimized linear regression, or using water peak non-linear parameterization [42], [43]. Such direct T2* estimates allow for localized and individually specific neurofeedback training, which is more physiologically plausible, and which extends beyond conventional neurofeedback techniques without direct T2* approximation. Another advantage of SE SVS acquisitions is that it allows for a reduction of specific absorption rate (SAR) levels, especially, as compared to SE EPI. This is particularly relevant for neurofeedback training studies, where individuals are sometimes being scanned repeatedly for many hours. The SAR also significantly limits the number of slices that can be acquired with SE EPI sequences, especially at ultra-high magnetic fields [35]. Localized SE SVS is not restricted by these limitations once the neurofeedback ROI has been defined.

Overall, SE SVS acquisitions might be beneficial for neurofeedback studies in that they allow for a direct and subject-specific T2*-based feedback signal [42], and in that they operate at lower SAR levels as compared to GE and SE EPI techniques. The possibility to simultaneously estimate T2* and T2 contrast [42] as well as reduced sensitivity to susceptibility-related signal loss of the SE techniques [35] provide an additional motivation to explore the SE SVS approach for neurofeedback. To shed light on these potential advantages, we for the first time provide a direct comparison analysis of R2* estimations based on GE EPI and SE SVS techniques at 3 T and at 7 T magnetic fields. Our analysis involved standard quality measures such as percent signal change (Δ%), contrast-to-noise ratio (CNR), and t-statistics for region-specific time courses during standard functional localizer runs and during neurofeedback runs. Notably, we did not compare signal quality between 3 T and 7 T magnetic field strengths, which have previously been thoroughly investigated for SE EPI as well as for GE EPI techniques.

## Methods

### Experimental Design

Two different groups of 7 healthy volunteers each were scanned on a 3 T scanner (4 male, 3 female, age 28±7 years) and on a 7 T scanner (6 male, 1 female, age 33±9 years). All participants were right-handed according to a minimal score of 6 on the Edinburgh Handedness Inventory [52]. Study protocols were approved by the Ethics Committees of the Medical Faculty of the RWTH Aachen University and of the University of Pennsylvania. All participants gave written informed consent and were paid an allowance at the end of their participation.

**Figure 1:**
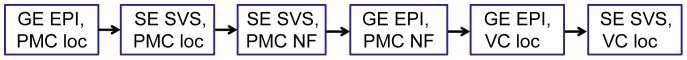
Sequence of data acquisition. GE – gradient-echo, EPI – echo-planar imaging, SE – spin-echo, SVS – single voxel spectroscopy, PMC – primary motor cortex, VC – visual cortex, NF – neurofeedback, loc – localizer.

The experimental protocol which was used to compare real-time GE EPI and real-time SE SVS acquisitions consisted of the 6 following runs (Figure 1): a GE EPI and a SE SVS based functional localizer of the primary motor cortex (PMC loc), a SE SVS and a GE EPI based neurofeedback run targeting the primary motor cortex (PMC NF), and a GE EPI and a SE SVS based localizer of the visual cortex (VC loc).

The primary motor cortex functional localizer runs consisted of finger tapping and baseline blocks, and the visual cortex functional localizer runs consisted of the presentation of a flickering visual checkerboard and baseline blocks. For the neurofeedback runs, the participants were instructed to adjust the speed and strength of their finger tapping so that a green horizontal bar would move up to the level of a predefined red horizontal target bar. All functional runs comprised 5 blocks of activation (i.e. finger tapping or visual stimulation, respectively), interleaved with 5 baseline blocks. Each block lasted 30 seconds, resulting in total run duration of 5 min.

### Data Acquisition

Functional GE EPI and SE SVS data were acquired on a 3 T and a 7 T MR scanner (Siemens Medical Solutions, Erlangen, Germany) equipped with a transmit body coil and a 12-channel phased array head receive coil at 3 T, or a birdcage single-channel head coil (quadrature) at 7 T. EPI images were obtained with a single-shot gradient-echo T2*-weighted sequence with 300 repetitions (TR = 1000 ms, 16 slices, volumes matrix size 64×64, voxel size = 3×3×3.75 mm^3^, flip angle α = 77°, bandwidth = 2.23 kHz/pixel, TE = 30 ms at 3 T; TE = 28 ms at 7 T). At 3 T, the water spectra were acquired using a spin-echo PRESS protocol with 300 repetitions (TE/TR = 30/1000 ms, flip angle α = 90°–180°–180°, bandwidth = 1 kHz, acquisition duration = 512 ms). At 7 T, the acquisition protocol was slightly different with TE = 20 ms, bandwidth = 2 kHz, acquisition duration = 256 ms. Spectroscopic voxels were chosen as isotropic as possible based on the individual GE EPI brain activation maps for the motor and visual conditions (approximately 1×1×1 cm^3^). On both scanners we performed a manual calibration of the transmitter amplitude and optimized the gradient shim currents using Siemens manual shimming adjustments in order to improve the spectroscopic signal quality. Acquisition parameters were selected to obtain a robust T2* estimate and the BOLD effect. Note that for the SE SVS pulse sequence, the TE was selected as short as possible for the given PRESS protocols, i.e. we targeted the T2* contrast. This was also done to reduce T2 weighting, and to acquire early-echo data for a more accurate T2* approximation. The SE SVS estimates of the T2* contrast were barely affected by the flip angle for the transversal magnetization. This was because the inversion pulses contributed to the T1 saturation, because the post-acquisition delay time was long compared to the T2 of the tissue, and because the T2* estimates were calculated from the free induction decay function (FID) directly. Neither water nor fat suppression was applied for the spectroscopy protocols. The first 10 acquisitions were discarded to avoid T1 saturation effects. The visual instructions and feedback were shown to the subjects via MR-compatible goggles (Resonance Technology Inc. Northridge USA) on the 3 T scanner, and projected to an MR-compatible screen on the 7 T scanner. The data were exported in real-time to the local PC and processed with the custom-made software as described in [43], [53].

### Data Processing and Feedback Signal Extraction

**Figure 2:**
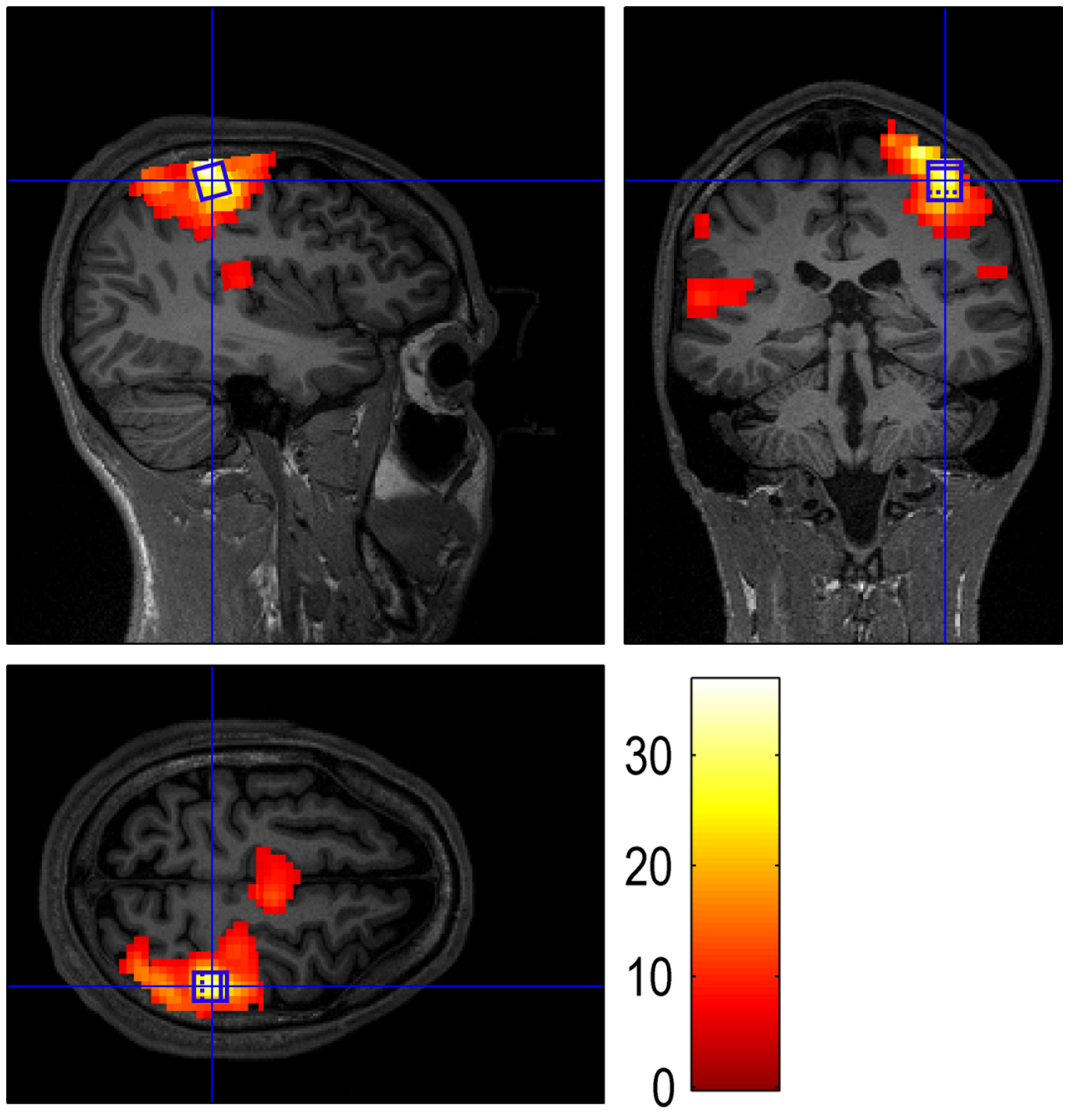
Illustration of the GE EPI and SE SVS ROIs. A GE EPI activation map and a single-voxel PMC ROI (blue) of a representative participant are shown on sagittal, transverse, and coronal planes of this participant’s structural scan. The PMC SE SVS ROI (size approximately 1×1×1 cm^3^) was defined to cover the voxels that exhibited a significant positive BOLD response to the left hand finger tapping. The GE EPI ROI was restricted to the SE SVS ROI.

Immediately after acquiring the data from the primary motor and visual cortex localizer runs, the images were pre-processed with SPM8 functions (Wellcome Trust Centre for Neuroimaging, Queen Square, London, UK), i.e. realigned to the first scan of the respective localizer run, and smoothed with an isotropic Gaussian kernel with 4 mm full-width-at-half-maximum (FWHM). Next, we specified a general linear model (GLM) with regressors for the experimental conditions, and acquired thresholded t-maps. For the SE SVS acquisitions, the single-voxel was defined so that it covered those voxels that exhibited a significant positive BOLD response to the left hand finger tapping or visual stimulation, respectively [43]. The ROI for the GE EPI acquisitions was restricted to the SE SVS ROI (Figure 2).

During the neurofeedback primary motor cortex GE EPI runs, the EPI volumes were first realigned to the first volume of the primary motor cortex functional localizer run. The feedback signal, which corresponded to the average activity within the ROI, was then calculated as soon as a new volume was acquired. During the neurofeedback SE SVS runs, the acquired water spectra were shifted to zero, filtered with a Gaussian filter, the eddy currents were compensated, and the water spectra were phase-corrected [54]. The feedback was provided after each FID acquisition as an absolute T2* measure which was estimated with the statistically optimized linear regression approach [43]. The optimal linear regression length was estimated based on the SE SVS primary motor and visual cortex runs. After the feedback signal was extracted from either SE SVS or GE EPI acquisitions, the signal was processed in order to reduce noise and to remove spike-like artifacts using our custom-made real-time software [53]. For the GE EPI acquisitions, the head motion parameters were taken into account, but head motion parameters were not available for the SE SVS acquisitions. To ensure that motion artifacts did not cause significant SE SVS signal distortions, we located relatively small ROIs within large active zones revealed by the primary motor and visual cortex localizer runs. Inter-run head movements between the SE SVS runs were controlled by acquiring GE EPI scans before and after the SE SVS runs; they were less than 1 mm.

### Time Courses Quality Measures and Comparison Analysis

**Figure 3:**
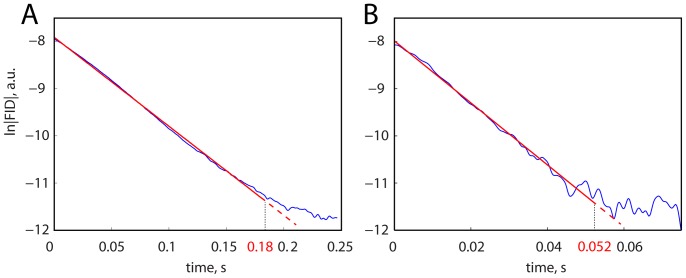
T2* approximation of the SE SVS data acquired at 3 T and at 7 T. The linear regression fits (red) are shown for single PMC ln|FID|’s (blue) for representative participants at 3 T (A) and at 7 T (B). Optimal linearization lengths are 0.18 s at 3 T (t = 41.6, p<0.001), and 0.052 s at 7 T (t = 16.7, p<0.001).

The comparison analysis between GE EPI and SE SVS acquisitions was based on their CNR, percent signal change, and t-statistics, and was performed separately for data acquired at 3 T and at 7 T. For SE SVS, statistically optimized linear regression was applied to the acquired FID in the time domain. The natural logarithm of the FID can be simplified assuming that the water signal is the dominating component in the acquired FID, and that all other proton sources of the signal are negligible [43]:
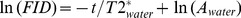
(1)with water time constant T2* and amplitude *A*. The linear regression was subsequently applied to the absolute logarithmic curve (*ln(|FID|)*) and determined by the optimal linear regression length (OLR) [43]. To compensate for line-broadening caused by applied Gaussian filtration, T2* estimation function was weighted with the correspondent filter coefficients [42]. The optimal linear regression length was estimated in the sense of a statistical measure, i.e. the maximum t-value in the distribution of t-values of time series computed for a set of linear regression lengths (Figure 3; red curves). The processed signal in Equation [1] may still have a large non-linear component because of the inadequate shimming conditions (Figure 3; blue curves), which can complicate the regression analysis of the acquired data. However, despite its simplicity, the proposed OLR approach has been shown to provide reliable T2* estimations at high and ultra-high magnetic fields [42], [43].

Because the GE EPI voxel intensity is proportional to exp(−*TE*⋅*R*2*), the T2* values estimated from SE SVS time courses were transformed to 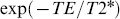 using the applied echo time *TE* and arbitrary scaling. This allowed for a direct comparison between GE EPI and SE SVS acquisitions.

Block-related averages were averaged across the time-course condition/baseline periods. The percent signal changes (Δ%) were estimated as an average from block-related condition/baseline 30-point plateaus. For the statistical analysis of the BOLD signal changes, we specified general linear models (GLM) with regressors for the experimental conditions defined in SPM8 (Welcome Trust Centre for Neuroimaging, UK). Each participant’s fMRI motion parameters were included into the GLM as nuisance regressors. Effects on the time-course quality ratings were analyzed in repeated-measures ANOVA for all data sets with functional *run* (PMC, PMC NF, and VC), MR *scanner* (3 T and 7 T) and acquisition *technique* (GE EPI and SE SVS) as within-subject factors. To further evaluate the difference between two samples, standard two-sample t-tests were used (*t*- and *p*- values; one-tailed). To calculate the CNR, we estimated differences between signal means during baseline and activation blocks, and their residual variances:
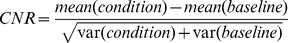
(2)where *condition/baseline* is the time course of the ROI in the functional localizer condition and baseline, respectively. All computations were carried out on a standard PC in Matlab 7.10 (The Mathworks, Natick, MA). The custom-made neurofeedback toolbox is available on request from the corresponding author.

## Results

**Figure 4:**
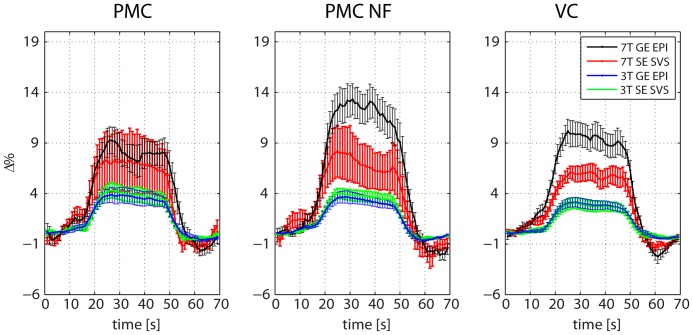
Block-related averages of the GE EPI and the SE SVS R2* time courses. Block-related averages were calculated for the 3 T GE EPI (blue), the 3 T SE SVS (green), the 7 T GE EPI (black), and the 7 T SE SVS (red) time courses of the PMC, the PMC NF, and the VC runs. Error bars represent the standard error of the mean.

To illustrate the quality of the acquired time courses, we evaluated the BOLD-dependent block-related signal changes in the GE EPI and the SE SVS time courses (Figure 4).

**Figure 5:**
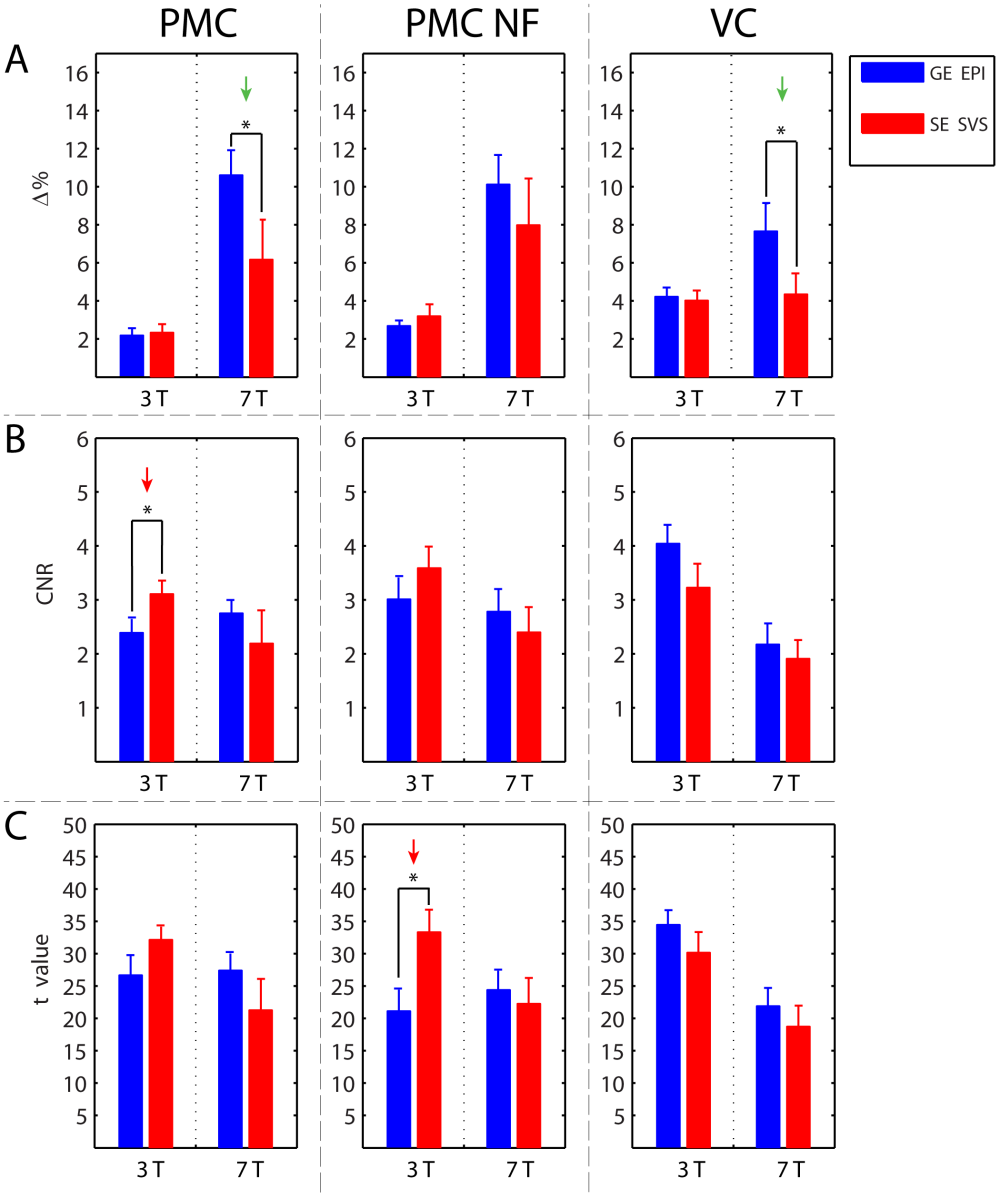
Performance comparison between GE EPI and SE SVS time courses at 3 We compared the time course quality of the PMC (1^st^ column), the PMC NF (2^nd^ column), and the VC (3^rd^ column) in terms of their percent signal change (Δ%; panel A), contrast-to-noise ratio (CNR, panel B), and t-statistics (t-value, panel C). This was done separately for data acquired with GE EPI (blue bars) and SE SVS (red bars), and separately for 3 T and 7 T acquisitions. At 3 T, the higher SE SVS CNR and t-value were indicated with red arrows. At 7 T, the higher GE EPI Δ% was indicated with green arrows. Error bars represent the standard error of the mean; asterisks denote statistical significance (p<0.05).

Overall, GE EPI and SE SVS acquisitions showed high data quality at 3 T and at 7 T in terms of the applied quality measures (Figure 5, see also Table 1 for numeric values). Compared to our previous study [42], where SE SVS time courses were estimated in terms of the T2* measures, R2*-weighting of the time-courses led to similar results. We found that the average R2* values in the PMC (18.9±0.1 s^−1^) and in the PMC NF runs (18.5±0.2 s^−1^) at 3 T, in the VC runs at 3 T (24.6±0.3 s^−1^), and in the VC runs at 7 T (55.9±0.5 s^−1^) were similar to previous findings [38], [32], [55]. However, the R2* values in the PMC (61.0±0.7 s^−1^) and in the PMC NF (59.8±0.9 s^−1^) runs at 7 T were somewhat higher. A repeated-measures ANOVA revealed a significant interaction of the factors scanner*technique (Δ%: F(1,12) = 5.7, p = 0.034; CNR: F(1,12) = 4.90, p = 0.047; t-statistics: F(1,12) = 5.7, p = 0.034). In addition, for percentage signal change, the interaction of the factors scanner*run was significant (F(2,24) = 4.1, p = 0.029). This implied that neither the data acquisition technique nor the field strength appeared to have an unambiguous advantage. Instead the performance depended on the specific combination of field strength, acquisition technique, and ROI. We therefore evaluated these factors using pair-wise comparisons.

**Table 1:** Percent signal change (Δ%), CNR, t-statistics for the GE EPI and for the SE SVS acquisitions at 3 T and at 7 T.

mean ± standard error		3 T			7 T		
		PMC	PMC NF	VC	PMC	PMC NF	VC
Δ**%**	**GE EPI**	2.2±0.3	2.7±0.3	4.2±0.4	10.6±1.3	10.1±1.5	7.7±1.4
	**SE SVS**	2.3±0.4	3.2±0.6	4.0±0.5	6.2±2.1	8.0±2.4	4.3±1.1
**CNR**	**GE EPI**	2.4±0.3	3.0±0.4	4.0±0.3	2.8±0.3	2.8±0.4	2.2±0.4
	**SE SVS**	3.1±0.2	3.6±0.4	3.2±0.4	2.2±0.6	2.4±0.5	1.9±0.4
**t-value**	**GE EPI**	26.7±2.8	21.1±3.2	34.4±2.1	27.4±2.5	24.4±2.8	21.9±2.7
	**SE SVS**	32.1±1.9	33.3±3.3	30.2±2.9	21.3±4.6	22.3±3.8	18.7±3.1

At 3 T, the GE EPI and the SE SVS acquisitions did not differ significantly in terms of Δ% for any of the runs (Figure 5A, PMC, PMC NF, VC: all t<0.8, all p>0.2). In contrast, at 7 T, Δ% was significantly higher for GE EPI acquisitions as compared to SE SVS acquisitions in the PMC (t = 1.8, p = 0.046), and in the VC runs (t = 1.9, p = 0.043). The same trend, albeit non-significant was evident also for the PMC NF runs (t = 0.8, p>0.2).

Comparing GE EPI and SE SVS acquisitions at 3 T in terms of their CNR (Figure 5B) showed significantly higher differences in the PMC runs (t = 2.1, p = 0.027), and smaller differences in the PMC NF (t = 1.1, p = 0.15) and in the VC runs (t = 1.5, p = 0.079). At 7 T, there was no significant CNR difference between the GE EPI and SE SVS acquisitions (PMC, PMC NF, VC: all t <0.8, all p>0.2). Notably, the SE SVS acquisitions at 7 T showed larger variations in the applied quantitative measures (Table 1).

The pattern of results for the t-statistics was similar to those of the CNR (Figure 5C), which is not surprising because they represent similar metrics. At 3 T, the t-values of the SE SVS acquisitions were significantly higher than those of the GE EPI acquisitions for the PMC NF runs (t = 2.7; p = 0.01), and higher for the PMC runs (t = 1.6; p = 0.067). No other differences between GE EPI and SE SVS acquisitions were found.

## Discussion

### GE EPI vs. SE SVS Analysis

We showed that real-time SE SVS at 3 T led to comparable data quality with that of real-time GE EPI. The BOLD percent signal changes for the GE EPI and for the SE SVS acquisitions at 3 T were stable, physiologically plausible, and did not differ between the acquisition methods (Figures 4, 5). Also, the CNR and the t-statistics of the GE EPI and the SE SVS acquisitions were comparable at 3 T. In some conditions, the SE SVS acquisitions at 3 T even showed enhanced performance compared to that of the GE EPI acquisitions considering the voxel size usually applied in real-time fMRI studies (Figure 5; red arrows).

At 7 T, the BOLD percent signal changes increased for both acquisition methods, but the signal changes of the GE EPI acquisitions were higher than that observed for the SE SVS acquisitions. Although our acquisition protocol was optimized for the T2* contrast, the estimation at 7 T was affected by the T2 contrast even at the shortest TE possible for the given MR sequence [39].

On the other hand, real-time SE SVS might allow for providing the T2 contrast at the same time as the T2* contrast in order to target specifically the microvasculature [37], [38], [42]. The decay rate of the FID is weighted by the intensity at time zero (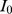), which is followed by the T2/T1 contrast: 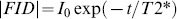. Note, that in our SE SVS pulse sequence, the SE data acquisition starts at time TE (i.e. time zero) and the percent T2 changes can be approximated while neglecting the T1 effect, i.e. 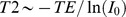 if 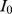 is given. Taking into account that the latter estimation was applied for a shorter than canonical spin-echo TE used for T2 contrast, and that it could be biased if the FID contains multiple components, the calculated percent signal changes values were ∼0.2% [42]. Note, that the present study was designed for an optimal T2* approximation and could be further balanced for higher T2 contrast, e.g. by using a larger TE [37], [38], [56], [57]. This supports that real-time SE SVS with longer TEs might allow for a neurofeedback signal originating from specifically the microvasculature T2 and T2* dynamics. Further research is needed to explore this possibility.

The Δ% estimated at 7 T confirmed that GE EPI yields a very high BOLD contrast at ultra-high field strengths. However, the advantage of ultra-high magnetic fields is reflected in improved CNR and improved statistics only if noise level can be controlled; particularly increased susceptibility artifacts, and local field inhomogeneity. In our study, the signal change increased at 7 T, but not the CNR and the t-statistics, which indicates that noise level increased as well. This is also illustrated by the increased variance of the R2* time courses (Figure 4). Also, whereas the average R2* values in the VC runs at 3 T and at 7 T, and in the PMC runs at 3 T were plausible, the R2* values in the PMC runs at 7 T were somewhat higher. The latter and the fact that the T2* approximations were more stable in the VC runs [42], [43] suggest suboptimal shimming in the PMC runs. Recently proposed local B_1_ (B_1_^+^) shimming in combination with a multichannel transceiver array coil might address this limitation [58]. Additionally, static magnetic field inhomogeneity (B_0_) for a single-voxel approach can be relatively easy compensated with a strong second-order and, if available, third-order shim system [47].

In our study, the difference between acquisitions did not only depend on the acquisition method, but was also region specific. For example, reduced CNR and t-statistics for the GE EPI runs at 7 T was also observed for the VC ROI, but not for the PMC ROI (Figure 5, Table 1). This might be due to the suboptimal shimming and, for SE SVS, the linearization length function (i.e. the individual T2*/R2* estimation), which can be very specific depending on the ROI and on the magnetic field strength [42], [43]. Interestingly, our GE EPI PMC and PMC NF runs benefit more from 7 T compared to the VC runs (Figure 5).

### Challenges and Benefits of the SE SVS Acquisitions

Given the potential advantage of SE SVS, such as a physiologically more plausible neurofeedback signal (via direct and subject-specific T2* estimation), SE SVS might be a suitable alternative to GE EPI. Scanning-extensive neurofeedback training studies have more pronounced SAR limitations, especially if fast protocols with repetition times (TRs) of less than 1 second are being used. In that case, localized SE SVS protocols might also be advantageous. Due to the joint signal acquisition and due to the fact that between-voxel averaging is not necessary, SVS at least theoretically might achieve better performance than GE EPI acquisitions. In our study, this might be reflected by higher SE SVS than GE EPI CNR at 3 T (Figure 5B). However, due to higher local field inhomogeneity, SVS is more vulnerable to partial volume artifacts at 7 T. As an alternative, multi-voxel spectroscopy approaches [59]–[64] provide spatially specific spectroscopy information of sufficient data quality, but at the expense of lower SNR compared to classical spectroscopy readouts [63], [65]. Also, the multi-echo GE EPI technique might be an efficient alternative to reduce some sources of artifacts for real-time imaging [66]. In addition, a direct voxel-wise estimation of T2* by using real-time multi-echo GE EPI protocols might also be possible. However, this has not been addressed so far and requires a thorough investigation especially at ultra-high magnetic fields where voxel-wise R2* approximation from 3–5 echoes could be compromised by high noise. The SE SVS technique uses the whole FID for such an approximation, and therefore allows for superior precision.

Furthermore, SE acquisitions allow for a T2 contrast which is particularly advantageous for limbic areas, e.g. the amygdala, where GE EPI fails to provide high signal quality due to susceptibility-related signal loss. Since SE acquisitions are less prone to such signal loss, they are of particular interest for neurofeedback studies targeting limbic areas.

## Conclusions

We evaluated the data quality of real-time GE EPI and SE SVS acquisitions in PMC, PMC NF, and VC runs. Overall, our results showed that data quality for these two acquisition methods is comparable at 3 T, and generally lower for SE SVS than for GE EPI at 7 T. Nevertheless, SE SVS acquisitions can be an interesting alternative to the GE EPI acquisition method for real-time applications. In particular, SE SVS allows for fast, direct and localized T2* estimation and thus a physiologically more plausible neurofeedback signal as compared to the methods that don’t provide a direct T2* estimation. Further, the SE SVS acquisition might allow for providing a combined T2* and T2 contrast, and thus potentially for targeting specifically the macro- and microvasculature, for reducing SAR for scanning-intensive neurofeedback training, and for reducing sensitivity to susceptibility-related signal loss in specific brain regions. However, these potential advantages need to be experimentally validated in future studies.
